# Coproduction, Coeducation, and Patient Involvement: Everyone Included Framework for Medical Education Across Age Groups and Cultures

**DOI:** 10.2196/31846

**Published:** 2021-11-03

**Authors:** Amy Price, Aishini Damaraju, Poorna Kushalnagar, Summer Brunoe, Ujwal Srivastava, Marcella Debidda, Larry Chu

**Affiliations:** 1 Stanford Anesthesia Informatics and Media (AIM) Lab Department of Anesthesiology, Perioperative and Pain Medicine Stanford University School of Medicine Palo Alto, CA United States; 2 University of Texas Houston, TX United States; 3 Center for Deaf Health Equity Gallaudet University Washington, DC United States; 4 Brown University Providence, RI United States; 5 Pulmonary Wellness Foundation New York, NY United States

**Keywords:** medical education, coproduction, public and patient involvement, education, patient, involvement, age, demographic, model, framework, culture, exploratory, engagement

## Abstract

Medical education, research, and health care practice continue to grow with minimal coproduction guidance. We suggest the Commons Principle approach to medical education as modeled by Ostrom and Williamson, where we share how adapting these models to multiple settings can enhance empathy, increase psychological safety, and provide robust just-in-time learning tools for practice. We here describe patient and public coproduction in diverse areas within health care using the commons philosophy across populations, cultures, and generations with learning examples across age groups and cultures. We further explore descriptive, mixed methods participatory action in medical and research education. We adopt an “Everyone Included” perspective and sought to identify its use in continuing medical education, citizen science, marginalized groups, publishing, and student internships. Overall, we outline coproduction at the point of need, as we report on strategies that improved engagement. This work demonstrates coproduction with the public across multiple settings and cultures, showing that even with minimal resources and experience, this partnership can improve medical education and care.

## Background

Co-creation and community nurture engagement to invite the public inside, so that they can influence and co-create healthcare, research and community. The public is the sensor that powers influence. The public can bring evidence into practice (Amy Price, 2014 [1])

It is time to use the growing public thirst for knowledge about health interventions to benefit public health and the continuing professional development of health professionals. This is already happening, as individuals who thought about how to meet a need have partnered with researchers to deliver 3D limbs, pancreatic cancer tests, inexpensive microscopes made of paper, and brain valves to relieve cranial pressure [1]. In this viewpoint, we provide a background of prior work in coproduction and patient involvement in health care. Examples of how such work applies to continuing professional development of health professionals are provided, along with summaries of some key insights to use in practice. Readers may find examples that worked for others in this paper. The examples of scaffolding and knowledge sharing might be adapted for use in new programs or to revise otherwise effective programs to feature more patient inclusion for education.

The patient is not an entity, but a person and that person can be a medical problem solver (Amy Price, 2014 [1])

## The Commons: An Opportunity for Coproduction

Health care literature is limited [2] as it tends to focus on patient values and experience [3] rather than on the active adoption, implementation, and application of medical education. Economists faced similar problems in their field as they considered how to distribute resources (the commons) with limitations in administration and in procuring labor and expertise. Ostrom and others define *the commons* as the cultural and natural resources accessible to all members of society, including raw materials such as air, water, habitable earth, information, and, specific for this paper, medical knowledge and production. These resources are common and not privately owned. Elinor Ostrom and Oliver Williamson are economists and Nobel laureates, whose research demonstrated that ordinary citizens are capable of managing and sustaining resources without outside control. They assert that coproduction occurs by combining professional expertise with the energy expenditure, wisdom, experience, and skills of end users [4] nurtured through the core standards of love, empathy, watchfulness, care, reciprocity, and willing instruction [5]. This does not imply equality in skills or ownership but rather equity in regard to respect, voice, and access to shared resources. In health care, as in life, partnerships are seldom equal, likewise with the distribution of skills and access to resources. Shared and equitable access to skills and knowledge in health care can multiply influence and scaffold community members to enrich commonly held resources (the Commons Principle). The examples in this paper employ such “*Commons Principles*” through coproduced real-world teaching at the point of care, training in empathy, and coproduced research, as stated by the Ostrom Law: “A resource arrangement that works in practice can work in theory” [6].

## The Commons: Basic Education and Scaffolding

As medical educators adopt the Commons Principles, they can also make use of technical scaffolding by deploying computers as expert learning reservoirs that learners can use to scaffold each other [7,8]. Scaffolding is described as a temporary structure used to support and protect others as it aids in the navigation, learning, construction, maintenance, or repair of a structure or system. The Hole-in-the-wall Education Limited (HiWEL) project used technical scaffolding to operationalize learning. Specifically, HiWEL placed computers inside selected village walls in rural India. Curious but previously illiterate children taught themselves and each other to read, and acquired math and science skills while exploring together through computers [9]. These examples might be applied to virtual community medical education in a pandemic and within communities where learners of all ages can work together to solve medical problems and to increase health literacy.

## Health Care Commons and Coproduction

Although coproduction in medical education is an emergent practice, in medicine more generally, the principles of the commons can be adapted through self-management and self-care initiatives in health care. Self-management is associated with reduced costs and increased quality of life. For example, patients remotely manage complex conditions such as diabetes [10], anticoagulation therapies [11], home kidney dialysis, tube feeding, pain pumps, thyroid care, and asthma [12]. These interventions are potentially lethal when misused, and yet results show patients and the public are competent partners with clinicians in their health care [12]. We argue that commons and coproduction principles can be strengthened through adopting an “Everyone Included” framework:

where everyone is trusted and respected for the expertise they bring, where openness and experimentation is the norm, people have personal ownership of health, individual stories have a global impact, and the patient voice and choice is a part of all stakeholder decisions

13

## Examples of Coproduction in Health Care

### Coproduction and Continuing Professional Development in Health Care

Continuing medical education (CME) consists of educational activities that serve to maintain, develop, or increase the knowledge, skills, and professional performance and relationships that a physician uses to provide services for patients, the public, or the profession. The content of CME is the body of knowledge and skills generally recognized and accepted by professions within the basic medical sciences, the discipline of clinical medicine, and the provision of health care to the public.

Patients have traditionally seen minimal involvement in the CME process as curriculum creators, educators, or participants. Although research has shown that patient inclusion does indeed enrich CME, many CME creators are still unsure as to *how* they might meaningfully engage with patients. In 2017, the Accreditation Council for Continuing Medical Education (ACCME) collaborated with Stanford Medicine X, a patient-inclusive health care innovation program at Stanford University, to create a set of design principles for patient engagement in CME.

The Stanford Medicine X design team worked directly with the president of the ACCME, Graham McMahon, to lay out a strategic plan for the design initiative. After defining the challenge, which was to make the CME process more inclusive of patients in an effective and meaningful manner, Medicine X organized a design workshop at the 2017 Medicine X | ED Conference.

A group of 50 providers, patients, educators, and health care administrators convened at Stanford University in April of 2017. During the half-day workshop, participants shared experiences, success stories, and challenges with CME. With the help of design facilitators, they documented key themes and future opportunities for success in CME.

Following the workshop, the Medicine X team documented, archived, and uploaded the thousands of insights generated from the workshop. The Medicine X team worked to consolidate and organize the contributions into insight statements or learning opportunities [13].

One recurring theme identified in the workshop was the affective component of fear as a roadblock to the involvement of patients in continuing professional development. When the participants looked at ways to mitigate such features, they reached a fascinating insight: the opposite of fear is not courage but curiosity. With this information, the team explored a novel design question: How might we foster a culture of curiosity in CME? Sifting through the thousands of notes from the workshop, they structured three key insights (Textbox 1):

- Curiosity: Medical students, patients, and providers are more likely to retain information if they are inherently curious about the material and each other.
- Safety: CME learning is more highly optimized when patients and providers have a safe space to learn from each other.
- Diversity: Patients and providers involved in CME are most engaged when learning from individuals from different backgrounds, cultures, expertise, and ages.

**Key learnings and insights from the Medicine X | ED Conference.**

**Build trust by cultivating empathy and shared vulnerability through the power of storytelling, and by permitting others to be human**

The team observed that the diverse group of patients, clinicians, researchers, and technologists found that storytelling is a potent tool to cultivate both empathy and shared vulnerability. The Stanford Medicine X program uses storytelling as a tool at their cross-stakeholder convenings. In the process of creating a safe space for storytelling, the team learned that storytelling creates a culture of openness, understanding, empathy, and shared vulnerability among the participants.

**Cocreate with patients early in the curriculum development process by actively involving them in the development, delivery, and assessment of continuing medical education**

The inception of the Stanford Medicine X conference, a convening run by the Medicine X program, exemplifies these principles. Medicine X began as a serendipitous conversation on Twitter between the Executive Director, Larry Chu, MD, and a Stanford Health Care patient, Hugo Campos. Their conversation and relationship in the planning of the first Medicine X conference matured into a culture of cocreation among patients, providers, educators, and all stakeholders in health care. Involvement of patients in the development of the curriculum and planning of convenings broadens the research agenda and increases the relevance of outcome measures that matter to patients [14].

The convenors worked in partnership with patients to identify emotional, physical, financial, and logistical barriers to participation through a wide variety of programs. The Medicine X ePatient Scholarship Program, accessibility surveys, and conference are all codeveloped with patients to correctly identify and address barriers to participation. These methods create equity by respecting patients as peers, and by acknowledging their contributions in ways that are meaningful to them.

At Medicine X, there is a concerted and deliberate effort to elevate respect hierarchies by hearing and applying the knowledge shared by patients across medical practice and by acknowledging patients’ time and effort. We explore ways to return value to patients for their contributions. In the past, we have found that thoughtful gifts of appreciation, acknowledgements of support, and other creative forms of gratitude have come a long way in making patients feel genuinely respected and valued. As others have noted, financial compensation to patients for their time is essential and complex [15].

It is vital to create a diverse, open, and welcoming culture by promoting diversity in continuing professional development activities. We note that diverse patient contributions require measures of inclusivity that maintain respect for all forms of diversity, some of which may include disability/ableism, gender nonconformity, race, and disadvantaged background, among others.

### Health Care Commons and Online Pulmonary Fibrosis

The Pulmonary Wellness Foundation assembled clinicians, healers, patient engagement experts, researchers, caregivers, patients, and patient advocates to develop “LIVE YOUR LIFE with Pulmonary Fibrosis, A Peer Support Program.” We offered the 8-session curriculum to 10 individuals living with pulmonary fibrosis (PF). The main objectives were to explore the physical and mental challenges imposed by PF and to form new leaders in the PF community. The community adopted the “Everyone Included” framework [13] for health care innovation, based on principles of mutual respect and inclusivity.

The program adopted psychological safety strategies to emphasize participants’ unique skills and viewpoints. This program implemented an empathic, lightly facilitated, hierarchy-flattened model with collaborative workshops sessions, alternated with sessions led by subject matter experts, which were attended by patients and experts for live questions and answers.

The program launched using the “Welcome, YOU matter!” package which contained an honor code, best practices for online support groups, biographies of facilitators, and a self-evaluating instrument. Before each session, they shared topics and objectives, a library of resources, and a summary of the previous session’s findings. Postprogram evaluations were distributed to measure learning. Participants shared the following key differentiators from other support initiatives: a space created for patients in collaboration with patients; prereadings and summaries of discussions helped with organization of thoughts in advance of each session as well as with the practice of new concepts after each session, once they were alone; the lightly moderated format allowed for spontaneity and authenticity, while brainstorming on potential solutions in a nonthreatening environment was uplifting and empowering.

The program resulted in the development of coproduced strategies to help problem solve PF-related struggles. The 2017 Ultimate Pulmonary Wellness online textbook [16] will be updated and published with coproduced program findings, practical experiences, and patient-led suggestions (Textbox 2).

**Key learnings and insights from LIVE YOUR LIFE with Pulmonary Fibrosis, A Peer Support Program.**

**Identify patient partners within your expert network community and seek their collaboration to coproduce shared resources**

Patient partners can be influential experts and allies to coproduce shared resources for health care professionals; they can identify the practical experiences and needs of patients, approaches for quality improvement, and key clinical program findings in professional development.

### Commons and Coproduction Among Deaf, Deafblind, and Hard of Hearing People

The deaf, deafblind, hard of hearing, and signing communities are marginalized from access to health-related resources. This marginalization drives health disparity among the deaf, deafblind, and hard of hearing populations. Coproduction and coeducation through commons resource-sharing principles can reduce disparity. Dr. Kushalnagar, a deaf individual who is a health researcher, and her team were inspired by the collective resource-sharing commons in the Everyone Included framework [13]. They engaged in several conversations with the leaders and emerged to engage deaf patients in coproducing research, storytelling videos, and more. The inclusion of deaf patients in public coproduction helps promote not only diversity in perspectives but also engagement in health-related decision-making between deaf patients and physicians [17,18].

The Everyone Included initiative increases visibility and accessibility to a diverse group of deaf, deafblind, and hard of hearing patients who can help, through user experience testing or human-computer interaction research, improve the digital health experience for everyone (Textbox 3). As we work to promote the Everyone Included model, the quality of health care research, health technology products, and patient experiences will improve. For example, Gallaudet University collaborated with Google to test and improve the user experience for its Google Live Transcribe speech-to-text app [19]. This app is now used by not only deaf people but everyone with a wide range of communication needs [20].

**Key learnings and insights for applying the Everyone Included principle among deaf, deafblind, and hard of hearing people.**

**Work with marginalized groups of people who have health disparities and include them in your coproduction process**

Marginalized people bring unique insights into the health care system. Including them in the coproduction of continuing professional development activities helps to improve health disparities in community health populations.

### Parkinson Disease and Intergenerational Coproduction

Summer Brunoe is one of our Stanford Science, Technology, and Medicine Internship (SASI) [21] alumni, who focused on the following question: Could intergenerational dancing impact those with Parkinson disease (PD) and older people? She participated in the undergraduate learning opportunity “Artists and Scientist as Partners” at Brown University, led by Rachel Balaban and taught by Julie A Strandberg. Dance for All People (DAPpers) [22] was created in 2013, inspired by the Dance for PD program developed by the Mark Morris Dance Group. DAPpers is designed for individuals with PD and other movement challenges. The classes provide placements where students can build intergenerational community and experience through the power of dance. The relationships formed are meaningful for all participants and reduce feelings of isolation and loneliness in both populations [22].

The students show up, dance, and get to know the people as individuals. Although COVID-19 restrictions went into place while Summer was taking this course, DAPpers chose to be resilient and they continued virtually. She found that the exposure, the need to adapt to meet online needs, and the requirement to listen brought unexpected opportunities to learn from vulnerable people. She notes communicating and learning together are actionable principles of empathy and empowerment (Textbox 4).

**Key learnings and insights from the Dance for All People program.**

**Including intergenerational participants in a professional development program reduces feelings of isolation and loneliness**

As the current COVID-19 pandemic increases social isolation of our health care populations, older populations are at increased risk for isolation. Including intergenerational participants in professional development programs can increase engagement and decrease feelings of social isolation and loneliness for both groups.

### Coproduction and Mentorship in Young People

SASI [21] brings together students from different backgrounds and assigns them to small groups led by SASI alumni who trained as Mentors-In-Residence (MiR). The program was developed by necessity due to COVID-19 withdrawals of face-to-face programs at Stanford and lockdown restrictions worldwide.

The MiRs were coached in the Everyone Included principles of coproduction [13]. MiRs were catalysts to ignite creativity and collaboration among SASI students. Before the start of the program, MiRs completed a workshop on how to provide feedback and be empathic. The training prepared them to communicate with students to mentor them throughout the program. Empathy can be somatic (sensory), affective (emotional), and cognitive (thinking); it is the quality that allows us to acknowledge and identify with the feelings, intentions, limitations, and beliefs of others, while maintaining a distinct sense of self [23].

MiRs found that the insights from older mentors with established careers and decades of practice would sometimes fall on deaf ears of young students. It was the MiR’s job to take the information given to them by the program leaders and alter it so that it could be appropriately understood by young students who were only beginning their careers. Furthermore, MiRs also oversaw and facilitated group interaction and worked with students to complete the program’s capstone projects. This form of mentorship involved daily check-ins on progress, office hours for questions, and simply providing an ear to listen to the students. Due to COVID-19 and lockdown restrictions, international students were often in difficult situations with regard to their finances and home lives. In addition to program leadership, it was a MiR’s role to simply listen to the students and work to find solutions to improve their program experience. Mentorship moves beyond defining career trajectories to encompass emotional support, encouragement, and guidance (Textbox 5). Students completed substantial projects, including prototypes for personal protective equipment infographics for school reopenings. They also prototyped new technology in Mercedes Benz cars to prevent child deaths, and developed, produced, and submitted videos to teach children healthy lifestyles; Sutter-Health published three of these videos [24].

**Key learnings and insights from the Mentors-in-Residence program.**

Continuing medical education can encourage mentoring and train multimedia production online by moving beyond defining career trajectories to encompass emotional support, encouragement, and guidance, and is applicable across age differences, groups, cultures, and countries.

### Coproduction and Mentorship in Professional Development

Ujwal Srivastava, who attended the inaugural SASI [21] session, valued hearing from patients, doctors and mentors; meeting new friends; and the opportunity to develop clinical and nonclinical medical skills. He shares:

The most important thing I learned as a SASI participant was the Everyone Included model for health care that promotes empathy, trust, and open communication between all stakeholders in health care.

The following summer, Ujwal volunteered as a teaching assistant for SASI, where he evaluated before-and-after changes in students during the SASI program. Specifically, he considered how empathy levels change in SASI students through coproduction. All SASI students complete a group capstone project in which they consult with an actual patient (remotely) about their medical condition, and they use this information to codesign solutions with them. Based on their interactions, patients were asked to evaluate each SASI participant’s empathy individually, using the validated Consultation and Relational Empathy (CARE) measure [25], in which scores can range from 10 to 50. Preinstruction, students averaged a 31.3 CARE score. By the second interaction, the CARE score was 36.3, and by the end of the SASI program, the average CARE score was 40.8. These findings show that preclinical students can increase empathy through coproduction (Textbox 6).

**Key Learnings and Insights from the Stanford Science, Technology, and Medicine Internship program.**

The process of including trained patient experts in a professional development program could remove hidden biases that professionals may have about the knowledge patients possess and can lead to more engaged and productive conversations. Such coproduction empowers patients to be cocreators in their treatment and transforms the patient-physician dynamic by producing innovative, holistic care.

## Conclusion

Many years ago, one of the authors (AP) worked as part of the Red Cross disaster team for Hurricane Andrew [26]. One of her tasks was to mark and report the dead and to mark properties with an “X” that were beyond reconstruction. It seemed so little. She learned that the most challenging emotion was not fear, anger, or sadness but rather helplessness, and the most destructive behavior was blame. Teams were admonished not to look back as other groups would follow to meet the needs for which they were not equipped. Relief teams were encouraged to focus on the living without respect to their current state or behaviors and to prepare them to receive benefit. Being stewards for the diverse patient communities and medical education networks, and preparing them to receive benefit is critical to maintaining the stability of health education, health technology, and health-related production work in empathy as a “public good” or “commons” for these communities, while at the same time creating research and commercial value that benefits Everyone Included.

In keeping with coproduction practice and the Everyone Included framework, we report that three of our authors (AB, SB, US) are students and members of the public one (PK) is a deaf researcher, and another researcher (AP) sustained disability and brain injury through trauma.

## Abbreviations

ACCME: Accreditation Council for Continuing Medical Education
CARE: Consultation and Relational Empathy
CME: continuing medical education
DAPpers: Dance for All People
HiWEL: Hole-in-the-wall Education Limited
MiR: Mentors-In-Residence
PD: Parkinson disease
PF: pulmonary fibrosis
SASI: Stanford Science, Technology, and Medicine Internship
